# Protic phosphonium-based ionic liquids for intermediate temperature polymer electrolyte membrane fuel cells

**DOI:** 10.1039/d6ra04431j

**Published:** 2026-07-23

**Authors:** T. Bertolin, V. Theußl, R. Leiritz, P. Schulz, C. Korte

**Affiliations:** a Institute of Energy Technologies – Electrochemical Processes Engineering (IET-4), Forschungszentrum Jülich GmbH 52428 Jülich Germany c.korte@fz-juelich.de; b Friedrich-Alexander University Erlangen-Nuremberg, Chair of Chemical Reaction Engineering 91058 Erlangen Germany; c RWTH Aachen University, Institute of Physical Chemistry 52074 Aachen Germany

## Abstract

Intermediate-temperature polymer electrolyte membrane fuel cells (IT-PEMFCs) with operating temperatures around 100 °C represent a future technology to circumvent problems arising in commercially available PEMFCs by simplifying water and heat management. Elevated temperatures necessitate electrolytes enabling proton conduction in anhydrous states. As such, ionic liquids, particularly protic sulfoalkylphosphonium-based ionic liquids (PPILs), emerge as promising candidates, due to their protonic conductivity at elevated temperatures and anhydrous conditions. Sulfoalkylphosphonium-based cations manifest high proton-donor activity, conferring Oxygen Reduction Reaction (ORR), as well as apolarity, allowing sufficient oxygen solubility, ensuring high transport-limited current density. Sulfoalkylphosphonium-based cations exhibit lower charge density and higher apolarity. In this study, six protic sulfoalkylphosphonium-based ionic liquids (PPILs) were investigated, combining two anions, trifluoromethanesulfonate [TfO] and bis(trifluoromethane)sulfonimide [TFSI], with three cations, tributyl(3-sulfopropyl) phosphonium [tBP], tributyl(4-sulfobutyl) phosphonium [tBB], and trioctyl(3-sulfopropyl) phosphonium [tOP]. The conducted measurements revealed higher oxygen solubility than for the N-analogue (10^−5^ mol cm^−3^*vs.* 10^−6^ mol cm^−3^ at 120 °C). [TfO]-based PPILs oxygen diffusivities are lower compared to the [TfO]-based N-analogue (10^−7^ cm^2^ s^−1^*vs.* 10^−5^ cm^2^ s^−1^ at 120 °C), but [TFSI]-based PPILs exhibited similar values. Altogether, higher limiting current densities for ORR can be measured (10^−1^ mA cm^−2^*vs.* 10^−2^ mA cm^−2^ at 80 °C). The thermal stability of [TfO]-based PPILs is sufficient for future IT-PEMFCs.

## Abstract 2

New protic phosphonium-based ionic liquids (PPILs) are investigated to improve the electrochemical performances of future intermediate temperature polymer electrolyte membrane fuel cells (IT-PEMFCs).

## Graphical abstract

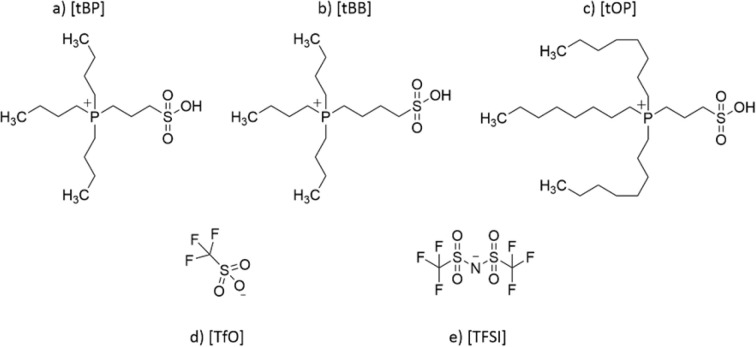

## Introduction

Polymer electrolyte membrane fuel cells (PEMFCs) have attracted attention in recent decades as a promising technology for producing clean energy by converting chemical energy into electricity. In addition, their ability to refuel the reactants, similar to internal combustion engines, further increases their viability as a vehicle drive system compared with rechargeable lithium batteries.^1^

The centrepiece of polymer electrolyte fuel cells is a proton conducting polymer electrolyte membrane (PEM). In already commercially available PEMFCs, either NAFION^®^ (*σ*_H^+^_ = 0.1 S cm^−1^, *x*(H_2_O) > 0.1 mol mol^−1^ and *T* > 50 °C) for temperatures below 80 °C or H_3_PO_4_/Polybenzimidazole (PBI) (PBI *σ*_tot_ = 0.02 S cm^−1^ at dry conditions and *T* = 140 °C) for high-temperature above 150 °C (HT-PEMFC) are available as membranes, dividing the operating temperature of the available PEMFC systems into two ranges.^2^ In the ionomer NAFION^®^, proton conduction is established by the high acidic sulfonic acid moieties and the presence of water in the polymer network. In the case of H_3_PO_4_/PBI the proton conductivity is established by the non-aqueous protic doping electrolyte H_3_PO_4_, exhibiting a very high autoprotolysis degree.

**Fig. 1:**
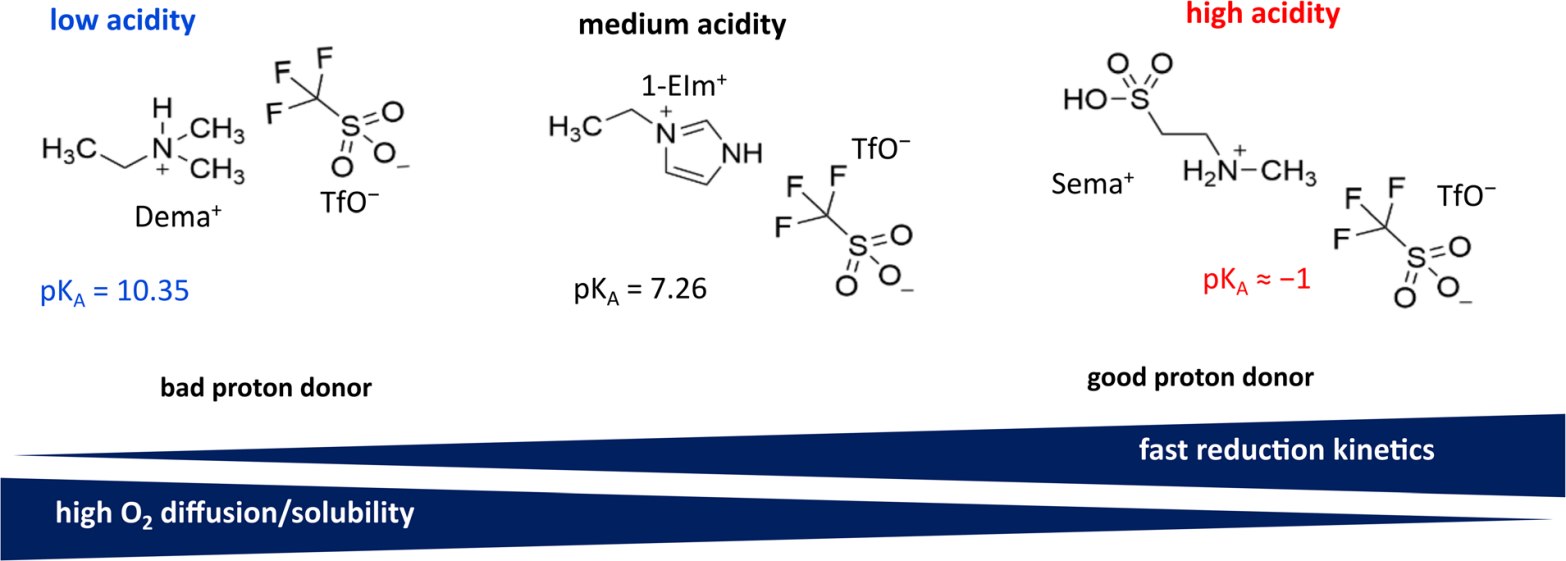
Trends regarding ORR kinetics and oxygen diffusivity/solubility of alkylammonium-/imidazolium-based PILs with various Brønsted acidity. [Dema][TfO]: diethylmethyl ammonium triflate, [1-EIm][TfO]: 1-ethylimidazolium triflate and [2-Sema][TfO]: 2-sulfoethylammonium triflate.

The main advantages of using elevated temperatures are simplified water management, enhanced waste-heat recovery, and decreased sensitivity of the Pt catalyst to impurities in the feed gas.^3^ However, the use of high-temperature PEMFCs based on H_3_PO_4_/PBI membranes is associated with low power density due to sluggish ORR kinetics caused by catalyst poisoning by anionic species of the phosphoric acid.^4^ For future intermediate/high temperature PEMFC operation to achieve the enumerated advantages, a new optimised non-aqueous yet protic doping electrolyte is mandatory.^2^ Protic ionic liquids are promising candidates for closing this “conductivity” gap. Ionic liquids (ILs) are typically composed of large organic cations and large anions, derived from superacids.^5^ The large, bulky cations and anions with low charge density result in low lattice energy in the solid crystalline phase, leading to exceptionally low melting points, often far below 100 °C. As molten salts, ILs provide ionic conductivity, combined with low volatility and high thermal and electrochemical stability. Generally, there are two classes: aprotic ionic liquids (AIL) and protic ionic liquids (PIL). For PIL, the cation (or the anion) exhibits an acidic protic functional group (Fig. 1). PILs with an acidic cation can be simply prepared by protonation (see eqn (1)) of an organic base (B, cation precursor) by a strong acid (HA, anion precursor):

1 HA + B → HB^+^ + A^−^

Thus, protic ionic liquids can act as proton conductors in a PEMFC when immobilized in an (ionogene) polymeric matrix. Depending on the water content and acidity, they can exhibit a vehicular or cooperative mechanism.

Due to the strong coulombic interactions between the ions, local ordering is also present in molten salts. In a time average, HB^+^ cations are preferentially surrounded by A^−^ anions and *vice versa*.^6^ In addition, specific interactions arise from the molecular structure of the large ions. Depending on the hydrophobic/unpolar and hydrophilic/polar properties of the ions, cations or anions can cluster, resulting in a mesoscale superstructure. There are also hydrogen bonds, A^−^⋯HB^+^, between cation and anion, leading to strong ion pairs. This may lead to a decrease in ionicity, an increase in viscosity, and a decrease in ion mobility and thus in conductivity.^7,8^ Important prerequisites for a protic electrolyte for future use in a fuel cell are fast ORR kinetics and thus low activation overpotentials; high oxygen permeability and hence low diffusion overpotential/high limiting current density; high proton conductivity; and sufficient thermal stability. Several PILs have already been investigated in the literature as possible proton-conducting electrolytes for PEMFCs at elevated temperatures. Watanabe *et al.* have investigated diethyl methylammonium triflate ([Dema][TfO]) and composite membranes of [Dema][TfO] with sulfonated polyimide (sPI) as host polymer. Thus, they found that these composite membranes exhibit high ionic conductivity and gas permeability comparable to hydrated NAFION^®^. Furthermore, they successfully operated a fuel cell with the prepared composite membranes under non-humidified conditions at a current density of 250 mA cm^−2^ at 120 °C, making them potential candidates for PEM fuel cells operating at elevated temperatures between 100–140 °C.^9,10^ In preceding own experimental studies, alkylammonium- and imidazolium-based PILs with varying cation Brønsted acidities were investigated for their electrochemical properties necessary for fuel cell operation, *i.e.* the oxygen reduction reaction (ORR) and the hydrogen oxidation reaction (HOR).^11–13^ Generally, ORR kinetics limit the overall power density of a PEMFC. It was found that especially PILs with high acidic cations show promising results regarding exchange current density and low overpotential, see Fig. 1.^14,15^

The limiting kinetic rate parameter of the ORR in the case of the high acidic sulfoalkylammonium-based PILs is about one order of magnitude higher compared to the low acidic alkylammonium- and imidazolium-based PILs. Furthermore, sulfoalkylammonium-based PILs reach higher ORR kinetic current density on platinum cathodes compared to phosphoric acid (H_3_PO_4_), as the [TfO]^−^ is less adsorbing on the Pt surface and thus blocks active sites.^16^ The ORR limiting current density depends on the limiting diffusional flux of oxygen to the electrode surface and thus on the product of the chemical diffusion coefficient and the equilibrium solubility of oxygen. Unfortunately, the oxygen diffusivity/solubility of the high acidic sulfoalkylammonium-based PILs is lower than that of the less acidic alkylammonium- and imidazolium-based PILs; see Fig. 1. The sulfoalkylammonium-based PILs [DEMSPA][TfO] (*N*,*N*-diethyl-*N*-methyl-3-sulfopropan-1-ammonium triflate) and [DESPA][TfO] (*N*,*N*-diethyl-3-sulfopropan-1-ammonium triflate) exhibit values only in the same order as 98% w/t H_3_PO_4_.^11^ The most commonly reported results on PILs for IT-PEMFC are based on ammonium-based PILs;^17–20^ however, sulfoalkylphosphonium cations (PPILs) are also potential candidates. A comparison between ammonium- and phosphonium-based protic ILs revealed that phosphonium-based PILs exhibited higher thermal stability, greater ionicity, and more facile proton reduction than their ammonium counterparts due to the lower basicity of the phosphines.^21^ Furthermore, the PPILs exhibit a larger, positively charged phosphorus atom, thereby increasing molecular volume. The higher polarizability of phosphorus than nitrogen enhances charge dislocation, and these effects should increase the affinity of molecular oxygen for the PPILs, as they are more apolar than the analogous ammonium-based PILs, thereby increasing ORR kinetics. The cations also have sulfonic acid groups; thus, their Δp*K*_a_ values should be about 17–18, allowing them to act as strong proton donors to maintain the ORR. A higher thermal stability than the ammonium-based PILs should also be present due to the high Δp*K*_a_ and high molecular weight. All these properties should indicate the material's suitability as an electrolyte for a future IT-PEMFC. To close this scientific gap, this research publication presents six novel phosphonium-based ionic liquids (PPILs) as electrolytes for the future intermediate-temperature polymer electrolyte fuel cell (IT-PEMFC). The PPILs consist of two different anions, triflate [TfO] and bistriflimide [TFSI], combined with six sulfoalkyl-substituted phosphonium cations: tributyl (3-sulfopropyl) phosphonium [tBP], tributyl (4-sulfobutyl) phosphonium [tBB], and trioctyl (3-sulfopropyl) phosphonium [tOP]. In these structures, the proton-donating functional group is not located on the phosphonium centre itself, but on the sulfoalkyl side chain (–SO_3_H). The main differences between the cations are the increasing molecular volume, weight, and alkyl chain length. To assess their suitability as electrolytes for the IT-PEMFC, the electrochemical and physicochemical properties at low water content (∼0.1 mol mol^−1^) are determined. For this, potentiostatic electrochemical impedance spectroscopy (PEIS) is used to determine specific conductivities, and, in combination with dynamic viscosity values, Walden plots are compiled to obtain information on the proton-conduction mechanism. Furthermore, electrochemical stability is assessed using linear sweep voltammetry (LSV), and the oxygen solubility and diffusion coefficient are determined by chronoamperometry (CA). To ensure thermal stability at elevated temperatures up to 250 °C, thermal gravimetric analysis (TGA) and attenuated total reflection infrared (ATR-IR) measurements were also conducted.

## Experimental part

### Preparation of PPILs

**Fig. 2:**
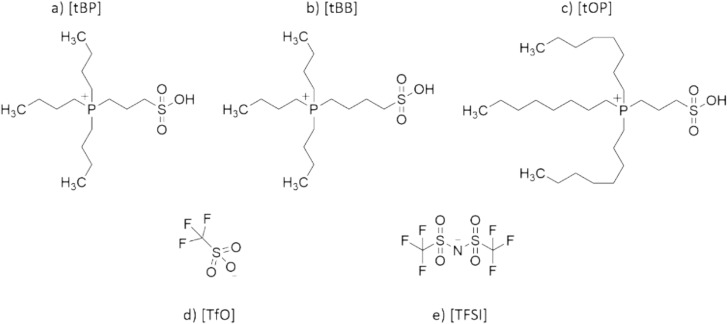
Molecular structure of the novel PPILs divided by cation (a–c) and anion (d and e). The cations are depicted from the lightest to the heaviest: [tBP]^+^, [tBB]^+^ and [tOP]^+^. The PPIL anions, [TfO]^−^ and [TFSI]^−^ are reported in the second row.

The novel PPILs are the combination of the three cations [tBP], [tBB], and [tOP] with two different anions [TfO] and [TFSI], see Fig. 2. The six novel phosphonium-based protic ionic liquids (PPILs) were synthesised by an S_N_2 reaction between a sultone and a trialkyl phosphine with various alkyl chain lengths. The phosphine (tri-*n*-butylphosphine/tri-*n*-butylphosphine/tri-*n*-octylphosphine, 1 eq.) was stirred with the corresponding sultone (1,3-propylsulton/1,4-butylsultone, 0.98 eq.) in acetonitrile at reflux temperature (82 °C) for three days. Diethyl ether was added to the mixture while stirring to precipitate a white solid, which was collected *via* filtration and cleaned with diethyl ether. To remove residual diethyl ether, the product was dried under vacuum. Adding a stoichiometric amount of the acid (trifluoromethanesulfonic acid/bis(trifluoromethanesulfonyl)imide) yielded the desired ionic liquid.^22^ The obtained PPILs were subjected to a drying process at 40 °C under vacuum (100 mbar) before each measurement. The water content of the PPILs was determined by Karl Fischer coulometric titration (852 KF Titrando Metrohm Deutschland GmbH & Co. KG, Germany), and the lowest obtained water content was around (0.1 ± 0.03) mol mol^−1^.

### Physical chemical characterization of PPILs

The density of the PPILs was measured with a Gay-Lussac pycnometer in borosilicate glass 3.3 (Brand GmbH + CO KG, Deutschland). The samples were measured over a temperature range of 50 to 130 °C in a dry-air atmosphere with a dew point of −(57 ± 1) °C. During measurements, a constant dry airflow was maintained above the pycnometer to keep the water content as low as possible. The samples were weighed with an ultra-microbalance (Mettler Toledo XP6U Excellence Plus). To determine the viscosity, a rheometer (HAAKE™ MARS™ Rheometer, Thermo Fisher SCIENTIFIC Inc., Germany) was used. The data was collected within a temperature range of 50–250 °C. The thermal stability of the new PPILs was investigated *via* thermal gravimetric analyses (TA TGA 550, TA Instruments WATERS™, USA) and infrared spectroscopy (Nicolet iS20 Spectrometer, Thermo Fisher Scientific Inc., USA). The measurements in the TGA were performed under a dry air atmosphere with a dew point of −(57 ± 1) °C in a platinum sample pan (volume: 100 µL) with a heating rate of 5 K min^−1^. To further investigate possible thermal degradation mechanisms of the novel PPILs, infrared analysis in the fingerprint region (500–1500 cm^−1^) was conducted. The PPILs were heated *via* a heating plate (GladiATR, PIKE Technologies Inc., USA) from 30 to 130 °C at a heating rate of 5 K min^−1^, and spectra were recorded every 10 °C.

### Electrochemical characterization of PPILs

For the electrochemical characterisation of the novel PPILs, a three-electrode set-up was employed, shown in Fig. S1. As working electrodes (WE), a Pt wire (radius 0.5 mm, length 6.3 mm, purity 99.9%, from Mateck Material, Technologie & Kristalle GmbH, Germany) and a platinum disc (radius: 0.125 mm, 99.9%, from Mateck Material, Technologie & Kristalle GmbH) were used, both embedded in glass jackets. A hydrogen-saturated palladium wire (PdH_*x*_) was used as a reference electrode (RE), and the reaction vessel, a cylindrical platinum crucible (*V* = 5 mL, 99.9% purity, m&k GmbH, Germany), was used as the counter electrode (CE). The PdH_*x*_ reference electrode has a stable potential of +50 mV *vs.* reversible hydrogen electrode (RHE) at 25 °C and is regenerated before each measurement.^23^ The Pt wire electrode setup has a defined geometric constant of (0.445 ± 0.004) cm^−1^ if the electrode is fully immersed in the electrolyte. A 0.01 M KCl aqueous solution is used for calibration. For more detailed information on the setup, the reader may refer to the existing literature.^16,24^ A potentiostat was used to apply defined potentials to the cell and to record the electrochemical data (SP-300, Bio-Logic SAS, France). For the preparation of the platinum electrode surfaces, a specific polishing procedure was implemented, starting with SiC 1200, SiC 2000 and finally SiC 4000 abrasive papers (Depki DP-Polishing Disc, Struers GmbH, Germany). On the polishing papers, a 0.3 µm and 0.05 µm alumina suspension (Micropolish Alumina, Buehler, Germany) was also applied. At the end of the polishing procedure, the electrodes were rinsed with distilled water and ethanol, then sonicated in Milli-Q water at room temperature for 10 min. The real electrode surface area (RSA) was determined by cyclic voltammetry in 0.5 M H_2_SO_4_ at room temperature, using the charge associated with underpotential deposition of hydrogen (HUPD). This yielded an RSA of (25.88 ± 0.01) mm^2^ for the Pt wire electrode and of (0.0673 ± 0.0001) mm^2^ for the Pt disc electrode.

The working electrode, reference electrode, and a glass tube to maintain a desired purge gas flow were inserted into a Teflon lid that closes the platinum crucible, which serves as the counter electrode. The cell setup was inserted into a metal vessel, heated by employing a thermostat for temperature control and a closed oil circuit for heat transfer (Lauda ECO Gold RE 415, LAUDA DR. R. WOBSER GMBH & CO. KG). The entire setup, including the metal heating vessel, was placed in a Faraday cage. The electrochemical stability of the novel six PPILs was investigated using linear sweep voltammetry (LSV) with a wire electrode setup. The experiments were conducted at temperatures between 80 and 120 °C, over a potential range of 1.2 V to −0.3 V *vs.* PdH_*x*_ and at a scan rate of 5 mV s^−1^. The current density was measured at 1 mV steps. The cell was flushed with dry N_2_ (gas flow: 15 mL min^−1^) at a constant temperature until a stable open-circuit voltage (OCV) was achieved. Thereafter, the LSV measurement was conducted. The oxygen saturation, solubility, and diffusivity were determined by potential-step chronoamperometry using a disk-electrode setup. Initially, LSVs were performed over a potential range of 1.2 V to −0.3 V *vs.* PdH_*x*_ and a scan rate of 5 mV s^−1^. The current density was measured at 1 mV steps. The cell was flushed with dry N_2_ (gas flow: 15 mL min^−1^) at a constant temperature until a stable OCV was achieved. The cell was also flushed with dry O_2_ (dew point: −57 °C ± 2 °C) at a constant temperature and a constant gas flow of 15 mL min^−1^ until a stable OCV was reached. Afterwards, an LSV was conducted to determine the ORR limiting current density range, which does not overlap with other reactions observed on the LSV under N_2_ gas. Afterwards, the potential was stepwise lowered from OCV to the ORR limiting current density range. The experiments were conducted at temperatures ranging from 50–130 °C. The obtained transient of the current density was analysed by utilising the Shoup–Szabo equation (see eqn (2)):

2 *I* = −4*FrD̃*_O_2__c_O_2__*nf*(*τ*)

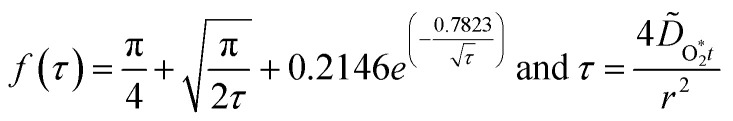


The Faraday constant is denoted by *F*, the RSA radius of the disk electrode by *r*, the chemical diffusion coefficient of oxygen by *D̃*_O_2__, the oxygen concentration by c_O_2__ and the number of transferred electrons during the ORR by *n* (*n* = 4). The total specific conductivity of the PPILs was determined *via* potential electrochemical impedance spectroscopy (PEIS) at 100 kHz and an amplitude of 5 mV at open-circuit voltage (OCV) using the Pt wire electrode. The measurements were conducted in a temperature range between 50 and 130 °C in an N_2_ atmosphere. The total specific conductivity for the PPILs was obtained from the ohmic resistance data and the geometric factor of the cell setup.

## Result and discussions

### Electrochemical stability

**Fig. 3:**
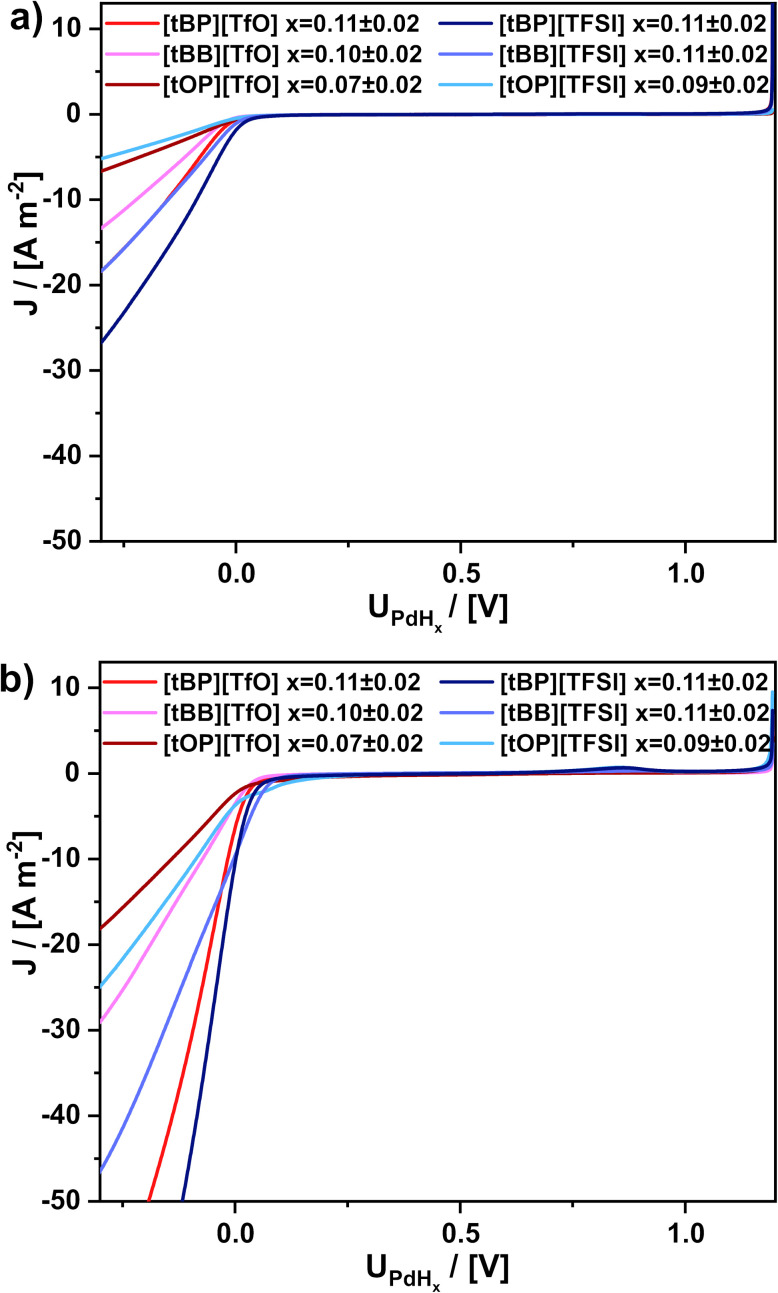
LSVs of PPILs conducted at different temperatures: (a) at 80 °C and (b) at 120 °C, measurement conditions: under N_2_ atmosphere with a scan rate of 5 mV s^−1^ [−0.25 V *vs.* PdH_*x*_; +1.2 V *vs.* PdH_*x*_].

An important parameter of an electrolyte suitable for operation in a fuel cell is its electrochemical stability over the potential range from 0 to 1 V *vs.* PdH_*x*_, *i.e.* the region between the hydrogen evolution reaction (HER) and oxygen reduction reaction (ORR). To determine the electrochemical stability of the novel PPILs, quasi-stationary Linear Sweep Voltammograms (LSV) were conducted at 80 °C (Fig. 3a) and 120 °C (Fig. 3b). Overall, all investigated PPILs have sufficient electrochemical stability. There are no indications for additional electrode processes. Regarding the influence of the cation on the HER-associated current densities, an inverse proportionality to the lengths of the alkyl chains is present, *i.e.* on the size of the cation independently of the chosen experimental temperatures and the present anion, as depicted in Fig. 3a and b for 80 and 120 °C. Table S1 additionally shows the radii of the investigated cations, underlining the influence of the cation size on the obtained HER-associated current densities. This is evident when comparing the measured current densities of the smallest and largest cations, [tBP] and [tOP] for *e.g.* 80 °C. At −0.2 V *vs.* PdH_*x*_ [tBP][TfO] and [tOP][TfO] show current densities of −12.89 A m^−2^ and −4.78 A m^−2^. [tBP][TFSI] and [tOP][TFSI] show, at the same voltage, −0.2 V *vs.* PdH_*x*,_ following current densities, −19.46 A m^−2^ and −3.70 A m^−2^.

In the investigated systems, the cations are the charge carriers that deliver the protons to the working electrode surface. When the potential drops to a sufficiently low level, approximately 0.1 V *vs.* PdH_*x*_, protonated species—namely sulfoalkylphosphonium cations and H_3_O^+^—accumulate from the bulk to the surface of the working electrode. Upon further lowering of the potential, they are reduced to adsorbed hydrogen atoms (H), which subsequently recombine and desorb as molecular hydrogen. The observed higher current densities for smaller cations can be explained by their higher packing density (coverage) at the electrode surface, which provides more protons available for reduction compared to bulkier cations. Based on the results, the HER current appears to be predominantly controlled by surface phenomena; smaller cations can deliver more protons to the electrode surface. As a result, this leads to higher current density for the hydrogen evolution reaction HER. Similar results have been obtained in a study about the influence of inorganic cations on the HER in aqueous electrolytes. It is reported that smaller cations occupy fewer surface-bound sites, also resulting in higher current densities.^25^

Regarding the influence of the anion on the HER, the [TFSI]-based PPILs exhibit, on average, only slightly higher current densities than the corresponding [TfO]-based PPILs, as depicted in Fig. 3a. This is evident when comparing [tBB][TFSI] and [tBB][TFO], as they exhibit at −0.2 V *vs.* PdH_*x*_ current densities of −12.91 A m^−2^ and −9.00 A m^−2^ at 80 °C, respectively. However, the spread of the conductivities due to the influence of the different cations is significantly higher than the influence of the chosen anion. Thus, this is not valid for all anion pairs with the same cation. A clear explanation for the anion influence cannot be given at this point.

### Oxygen solubility

**Fig. 4:**
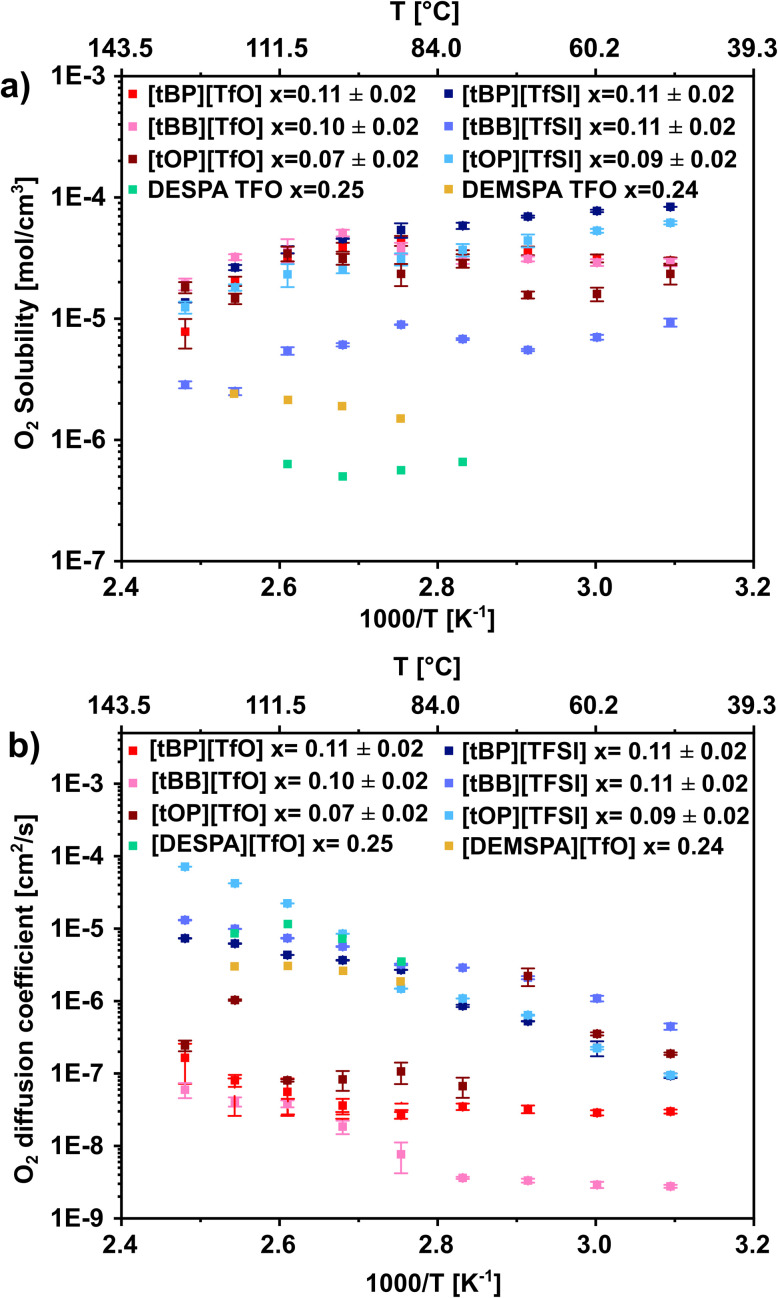
Logarithmic plot of oxygen properties within the investigated PPILs as a function of the temperature: (a) saturation concentration of molecular oxygen and (b) oxygen diffusion coefficients of oxygen along with the corresponding data for [DEMSPA].

When establishing novel PPILs as electrolytes for future PEMFCs, oxygen solubility and the oxygen diffusion coefficient are pivotal parameters to investigate, as the total amount of oxygen at the cathode needs to remain steady for fuel cell operation. Consequently, an increase in these parameters will increase the current density, thereby improving the performance of the PEMFC. The oxygen solubilities and the oxygen diffusion coefficients were acquired concurrently *via* chronoamperometric techniques over the temperature range of 50–130 °C. Fig. 4a illustrates the oxygen solubility across the aforementioned temperature range. With increasing temperature, the oxygen solubility decreases by two orders of magnitude (10^−4^ to 10^−6^ mol cm^−3^). This phenomenon could probably be attributed to the exothermic nature of oxygen dissolution within the PPILs, wherein elevated temperature weakens intermolecular interactions between the constituent ionic substrates and oxygen, thereby diminishing solubility. In addition, the decrease in solubility can also be linked to the unfavourable entropy change associated with gas dissolution, which becomes more significant at higher temperatures. The hypothesis posits that London dispersion forces predominate in oxygen dissolution, given the apolar nature of molecular oxygen. As reported in the literature, computational data confirm that van der Waals intermolecular forces contribute more significantly to oxygen solvation than electrostatic interactions. Consequently, as temperature increases, these van der Waals interactions weaken, resulting in a decrease in solvation.^26–28^

A closer examination of the influence of cation size on oxygen solubility reveals no discernible trend. The exception here are the [tBP]-based PPILs, as they exhibit enhanced oxygen solubility at temperatures below 90 °C. For instance, at 90 °C, the [tBP][TfO] and the [tBP][TFSI] exhibit oxygen solubilities of (4.4 ± 0.4) × 10^−5^ mol cm^−3^ and (5.38 ± 0.7) × 10^−5^ mol cm^−3^, respectively. One potential explanation for this phenomenon is that the [tBP] cation, due to its relatively small ionic radius, may possess a less shielded positive charge. This could result in a stronger dipole-induced dipole interaction than that observed for the [tBB] and [tOP] cations. Another contributing factor might be the dipole–quadrupole interaction between the cation and molecular oxygen. The [tBP] cation is distinguished by having the shortest alkyl chain (propyl) attached to the sulfonic acid group, as well as the shortest alkyl groups surrounding the phosphonic group (butyl), among those examined in this study. This suggests that its dipole is stronger and less shielded than those of [tBB] and [tOP] at low temperature. [tBP] dipole may interact more strongly with the oxygen quadrupole. This interaction is not as pronounced for the [tBB] and [tOP] cations, nor for the [tBP] cation at temperatures above 90 °C, where it is presumed that the higher conformational flexibility tends to decrease the dipole, leading only to London dispersive force interactions. Similar results have been reported in the literature for the oxygen solubility. It has been reported that, by considering the conformational flexibility of the alkyl chains with ether groups attached to the positive charges of pyrrolidinium, pyrimidinium and piperidinium-based ILs, the more flexible the alkyl chains are, the more they can bend toward the positive charge and the more they can shield its punctual electric field intensity.^26,27^ It can be concluded that the presence of a large alkyl phosphonium group in the cation substrates enhances van der Waals intermolecular forces. This enhancement results in a higher affinity for nonpolar molecules, such as molecular oxygen.

Considering the influence of the anion on oxygen solubility, the [TFSI]-based PPILs exhibit a constant decrease in oxygen solubility as temperature increases. For example, at 80 °C, [tBP][TFSI] exhibits an oxygen solubility of (5.8 ± 0.3) × 10^−5^ mol cm^−3^, whereas at 130 °C, it shows an oxygen solubility of (1.36 ± 0.1) × 10^−5^ mol cm^−3^. This trend indicates that the London dispersion forces between the [TFSI]-anion and oxygen most likely lead to an exothermic solvation of molecular oxygen. The hydrophobicity of [TFSI], mainly due to the presence of trifluoromethyl groups, indicates substantial delocalisation of negative charge and a highly symmetrical Cn geometry, leading to London dispersive forces. As reported in the literature, the [TFSI] anion, studied in imidazolium-based ILs, could transfer its negative charge to the oxygen atoms of the sulfonyl groups, thereby increasing their hydrophobicity.^29,30^ As temperature increases, the intensities of the London dispersion forces decrease, leading to lower oxygen solubility. For [TFO]-based PPILs, however, the London dispersive forces are less present as the [TfO] anion possesses a *C*_3v_ geometry with less effective charge delocalisation and stronger electrostatic and polar interactions. It may be that the [TfO] anion interacts less strongly with molecular oxygen *via* London dispersion forces, resulting in a less pronounced temperature-dependent change in behaviour than [TFSI]. As established in the extensive literature on the subject, the initial computation of molecular electrostatic potentials (MESP) indicates that the [TfO] anion tends to maintain a negative charge on the oxygen atoms, accompanied by slight delocalisation on the fluorine atoms. This observation supports the hypothesis that the [TfO] anion exhibits poor charge delocalisation, thereby decreasing hydrophobicity.^31^

In Fig. 4b, the diffusion coefficient of molecular oxygen for the six PPILs is shown to increase at higher temperatures. For example, [tBB][TfO] exhibits a diffusion coefficient of (3.6 ± 0.1) × 10^−9^ cm^2^ s^−1^ at 80 °C and one of (6.0 ± 1.4) × 10^−8^ cm^2^ s^−1^ at 130 °C, respectively. This common trend among the PPILs is related to the fact that, as temperature increases, the density and viscosity of the samples decrease (see Fig. S2a and c). With lower friction between the flowing molecules and more free volume among the ions, the molecular oxygen can move faster between the ions. This behaviour is well documented in the literature, *i.e.* the diffusivity of a gas within an IL is proportional to the inverse square root of viscosity (*D* ∼ *η*^−0.5^).^12,32,33^

By considering the cation influence on the oxygen diffusion coefficient, no particular relation exists, similar to the results about the oxygen solubility above. It might be deduced that the cation interacts mainly with molecular oxygen through London dispersion forces, resulting in high overall oxygen solubility regardless of ionic radius. On the other hand, the influence of the anion is more prominent. The [TFSI]-based PPILs present higher oxygen diffusion coefficients than [TfO]-based PPILs. A main role could again be the lower viscosity of the [TFSI]-based PPILs, as shown in Fig. S2c, which exhibit a lower dynamic viscosity than the [TfO]-based PPILs, leading to faster and more favourable mobility of the oxygen molecules. On the other hand, the [TfO]-based PPILs rely more on strong ionic interactions, leading to higher viscosity and a slower temperature-dependent increase in viscosity, resulting in higher friction and less free interionic volume for the oxygen molecules. Also, since the [TfO] anion relies more on these strong ionic interactions, the diffusivity, as mentioned before for the solubility, is less affected by temperature changes since they do not interact as much as the more apolar [TFSI] anion with the molecular oxygen.

Comparing the six novel PPILs to their ammonium-based counterparts generally reveals a higher oxygen solubility in the PPILs. This is particularly evident in the comparison of oxygen solubility at 100 °C, where [tBB][TfO] shows a solubility of (5.1 ± 3.2) × 10^−6^ mol cm^−3^. In contrast, [DEMSPA][TfO] and [DESPA][TfO] exhibit oxygen solubilities of (5.6 ± 0.1) × 10^−7^ mol cm^−3^ and (2.0 ± 0.1) × 10^−7^ mol cm^−3^, respectively, at the same temperature (see Fig. 4a for data for [DEMSPA][TfO] and [DESPA][TfO]).^11^ These higher values might be caused by larger polarizability and, as a consequence, more intense van der Waals forces. Substituting a phosphorus atom for the nitrogen atom in the positively charged core of the cations has been shown to enhance these characteristics. However, the lengths of the alkyl chains do not influence the oxygen solubility by increasing the strength of the intermolecular forces. On the other hand, the oxygen diffusion coefficient of the phosphorus-containing PPILs does not show higher values than [DEMSPA][TfO] and [DESPA][TfO]. The oxygen diffusion coefficients of PILs appear to be particularly dependent on viscosity, as proved by the data of the [TFSI]-based PPILs, which exhibit dynamic viscosity and oxygen diffusion coefficients comparable to those of [DEMSPA][TfO] and [DESPA][TfO]. In contrast, [TfO]-based PPILs have higher dynamic viscosity and lower oxygen diffusion coefficients than [DEMSPA][TfO].^11^

### Conductivity and Walden plot

**Fig. 5:**
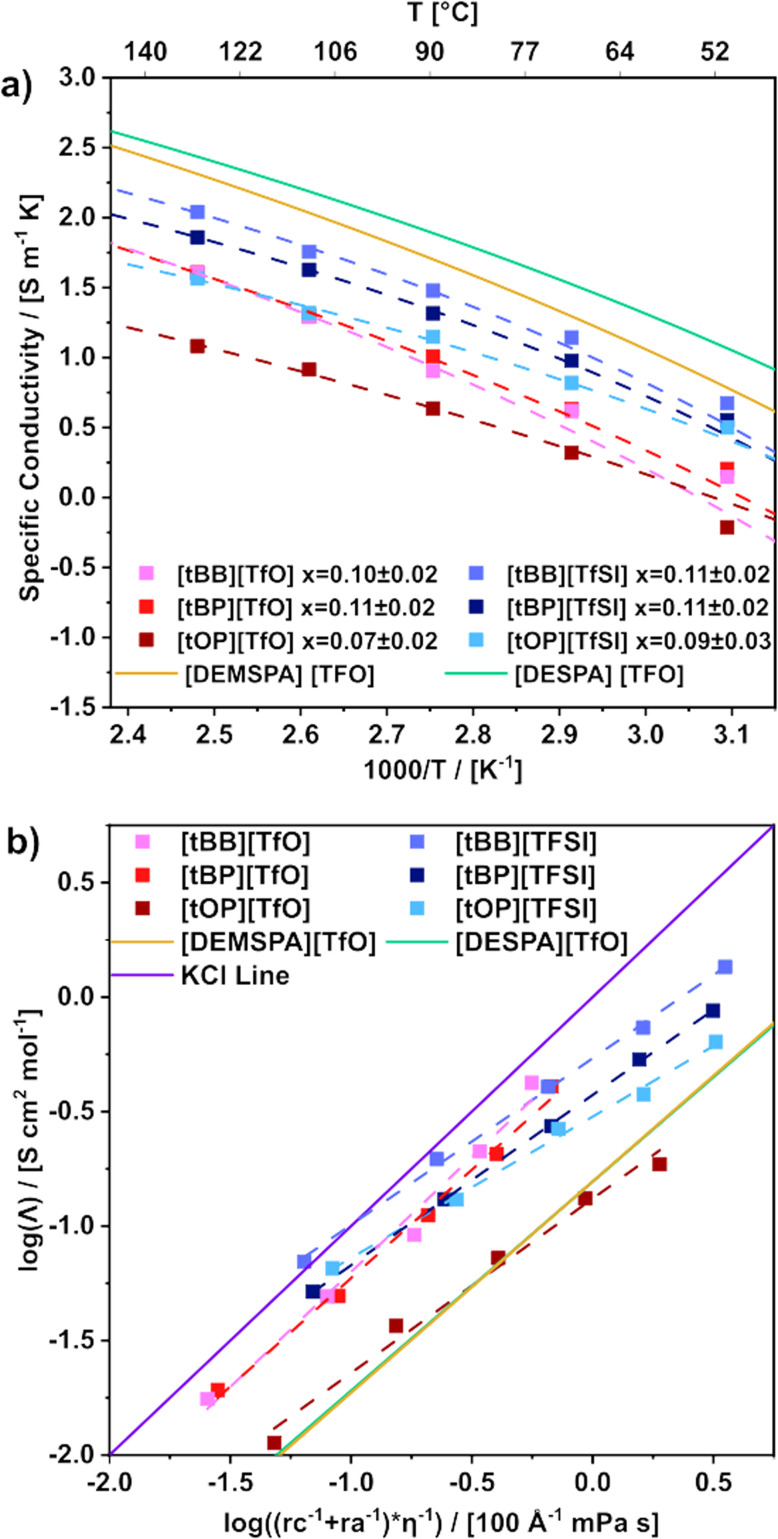
(a) Specific conductivity of the novel PPILs as a function of temperature with the corresponding VFT plots. (b) Radius-corrected Walden plot for the new PPILs, along with the corresponding lines for [DEMSPA][TfO], [DESPA][TfO]^9^ and the KCl line. The molar conductivity is obtained from the total specific conductivity of the PPILs, considering their water concentration.

The specific conductivity of the PPILs was measured to obtain the data required for the Walden plot to determine the conduction mechanism. The data were obtained from PEIS investigations at high frequency (100 kHz) and analysed as a function of temperature, between 50 °C and 130 °C, using the Vogel–Fulcher–Tammann equation (see eqn (S4)). As the temperature increases, so does the specific conductivity (Fig. 5a). For example, at 50 °C [tBP][TfO]exhibits a conductivity of (1.1 ± 0.1) × 10^−2^ S m^−1^, whereas at 130 °C it displays a conductivity of (1.7 ± 0.01) × 10^−1^ S m^−1^. This effect is consistent with eqn (S2) and (S4) in the SI, which provide a more detailed explanation of ion conduction. At high temperatures, the diffusion coefficients of ions in PPILs increase, leading to enhanced diffusion and higher conductivity when an external electric field is applied to the electrolyte. Further evidence of this is shown in the dynamic viscosity data in Fig. S2c. It is observed that the dynamic viscosity decreases as temperature increases. Considering eqn (S3), as the dynamic viscosity decreases, the specific conductivity increases. It can then be assumed that the ions contribute to the conductivity *via* a vehicular mechanism. As documented in the literature, in ILs, the network formed by the positive and negative charge distributions and its derived viscosity plays a crucial role in the vehicular conductive mechanism.^34^ Concerning the impact of the cation on the specific conductivity, it is possible to observe that the [tOP]-based PPILs manifest the lowest conductivity values. This effect might be understood by examining the trend of the dissociation parameter *α*_D_. The dissociation parameters *α*_D_ are derived from eqn (S7) through the comparison of dynamic viscosity and specific conductivity. The correlation between higher *α*_D_ and higher conductivity is attributable to the fact that more dissociated electrolytes tend to have higher ion mobilities and a greater number of charge carriers per mole of electrolyte (see Table S2). As demonstrated in the literature, ILs in aqueous solutions exhibit a strong correlation between ionic dissociation and overall conductivity, thereby substantiating the relationship between these parameters.^35,36^ Secondly, an analysis of the data reveals that among the PPILs sharing the same anion, the lowest conductivities are exhibited by those with the [tOP] cation. This phenomenon could be attributed to steric hindrance and alkylic interionic interactions of the largest cation, [tOP]. The long alkyl chains may interact with one another, thereby forming cationic domains within the PPIL and consequently reducing the overall ionic mobility. For a more detailed understanding, it is necessary to evaluate the Vogel–Fulcher–Tamman (VFT) parameters for the specific conductivity data. The VFT parameters B*σ* for [tOP][TfO] and [tOP][TFSI] (see Table S2) show the lowest values among the PPILs with common anion. Furthermore, the *T*_0_ values for [tOP][TfO] and [tOP][TFSI] (see Table S2) are the lowest recorded for the corresponding anion-based PPILs. These low values may be indicative of the maintenance of ionic mobility even at low temperatures, since [tOP]-based PPILs do not glaze easily due to weaker ionic interactions and less effective packing than the ones corresponding to [tBP] and [tBB]-based PPILs. However, as demonstrated by analyses of the dissociation parameters, the [tOP]-based PPILs exhibit the lowest *α*_D_ values (see Table S2). This indicates that [tOP]-based PPILs tend to form ion aggregates within the PPIL bulk. It could be that these ion aggregation processes comprise only the aforementioned cations. The existence of these domains has been comprehensively documented in the extant literature, and their increased prevalence in ILs with large hydrophobic cations has also been reported.^34,37,38^ It can be concluded that cations with large alkylic substrates lower the conductivity of PPILs due to the formation of large cationic domains.

By observing the anion influence, all [TFSI]-based PPILs exhibit higher conductivities compared to [TfO]-based PPILs. Since the [TFSI] anion can delocalize the negative charge more efficiently than the [TfO] anion, it might lead to weaker interionic attractive interactions. This hypothesis is further substantiated by the observation that all [TFSI]-based PPILs exhibited lower dynamic viscosities and, consequently, higher ion mobility, as evidenced by the dynamic viscosity results. As shown in Table S2, the assigned activation energies are lower for the [TFSI]-based PPILs than for the [TfO]-based PPILs, proving that the vehicular conductivity mechanism is faster. The weaker interactions could also be related to the less polar character of the [TFSI] anion than the [TfO] anion, which decreases the interaction strength with the surrounding anions. This effect would lead to a more dissociated liquid structure, as evidenced by all *α*_D_ values for the [TFSI]-based PPILs exceeding 0.7 (see Table S2). As reported in the literature, the more polarisable the ions, the more they tend to dissociate.^39–41^ These results are also in agreement with the results already shown in Fig. 3b. To conclude, we can assume that PPILs exhibit higher conductivities if their ion pairs are decoupled. In Fig. 5a, it is possible to compare the PPILs' conductivity values with those belonging to the ammonium-based PILs [DEMSPA][TfO] and [DESPA][TfO]. The novel PPILs exhibit comparatively lower conductivities. It can be postulated that the larger cations in PPILs exhibit reduced mobility in response to external electric fields compared with their ammonium-based PIL counterparts. A considerable difference between the total conductivity and the proton partial conductivity has to be taken into account. By considering the literature data for the high acidic ammonium-based PIL 2-sulfoethylmethylammonium triflate ([2-Sema][TfO]), which exhibits a proton transference number *t*_H^+^_ of 0.52 at low water content, it can be assumed that for the investigated PPIL [tBP][TfO] with the smallest cation, *t*_H^+^_ is likely in the same order but not higher. PPILs with an even higher cationic molecular mass will definitely have a significantly smaller *t*_H^+^_. At low water content, the vehicular transport mechanism remains dominant in the investigated PPILs.

From the logarithmic plot of the molar conductance (*Λ*) and the radius-corrected reciprocal dynamic viscosity (*r*_c_^−1^*η*^−1^+ *r*_a_^−1^*η*^−1^), the Walden plot for the novel PPILs (Fig. 5b) is obtained. From the linear fitting of the data, the log *C*′ and the *α* parameter were acquired (can be seen in Table S3). All the novel PPILs lie below the KCl line, and it is noticeable that the more the sample is dissociated, in accordance with the *α*_D_ parameter (see Table S2), the closer its line is to the KCl line. This parameter is related to log *C*′; the more negative the latter is, the less the ions are dissociated. Along with the previous considerations on conductivity, this indicates that the conductivity behaviour is strongly related to ionic dissociation, *i.e.*, the more ions are dissociated, the more they conduct *via* a vehicular mechanism, given the same amount of moles. It is evident from consideration of the cation influences that the [tOP]-based PPILs are positioned at the lowest positions of the Walden plot, exhibiting the most diminished values of log *C*′ (refer to Table S3). As the cation radius increases, PPIL dissociation diminishes. This observation is consistent with the earlier finding regarding their *α*_D_ values, which indicate a lower degree of dissociation. Furthermore, [tOP][TfO] and [tOP][TFSI] are the PPILs that show the lowest values of the *α* parameter (see Table S3), indicating that they manifest the lowest ionicity. This might be related to the fact that [tOP][TfO] and [tOP][TFSI] exhibited the lowest values of (*r*_a_/*r*_c_) ratio, suggesting that packing is probably unfavourable due to the different sizes of the ions and favouring the formation of cation aggregates. The less steep slope of the lines corresponding to the [tOP]-based PPILs, compared to those of the [tBB]-based and [tBP]-based PPILs, may be attributed to the formation of cation aggregates. As reported in the literature, cations constrained within a large alkylic matrix exhibit reduced mobility rather than a cooperative conduction mechanism.^42–44^ To confirm this hypothesis, further viscosity analyses at different shear rates are required.

With regard to the anion influence, it can be observed that the three [TFSI]-based PPILs present higher values of log *C*′, indicating a higher ionicity. Furthermore, the slopes associated with the [TFSI]-based PPILs are less steep than those observed for the [TfO]-based PPILs (see Table S3). This outcome could be related to non-Newtonian behaviour in [TFSI]-based PPILs, which will be confirmed by future analyses. In conclusion, the novel PPILs exhibit ionic behaviour across the entire temperature range investigated, with the [TFSI]-based PPILs demonstrating notably higher ionicity. Additionally, all novel PPILs display greater ionicity compared to the ammonium-based PILs, [DEMSPA][TfO] and [DESPA][TfO].^11^

### Thermal stability

**Fig. 6:**
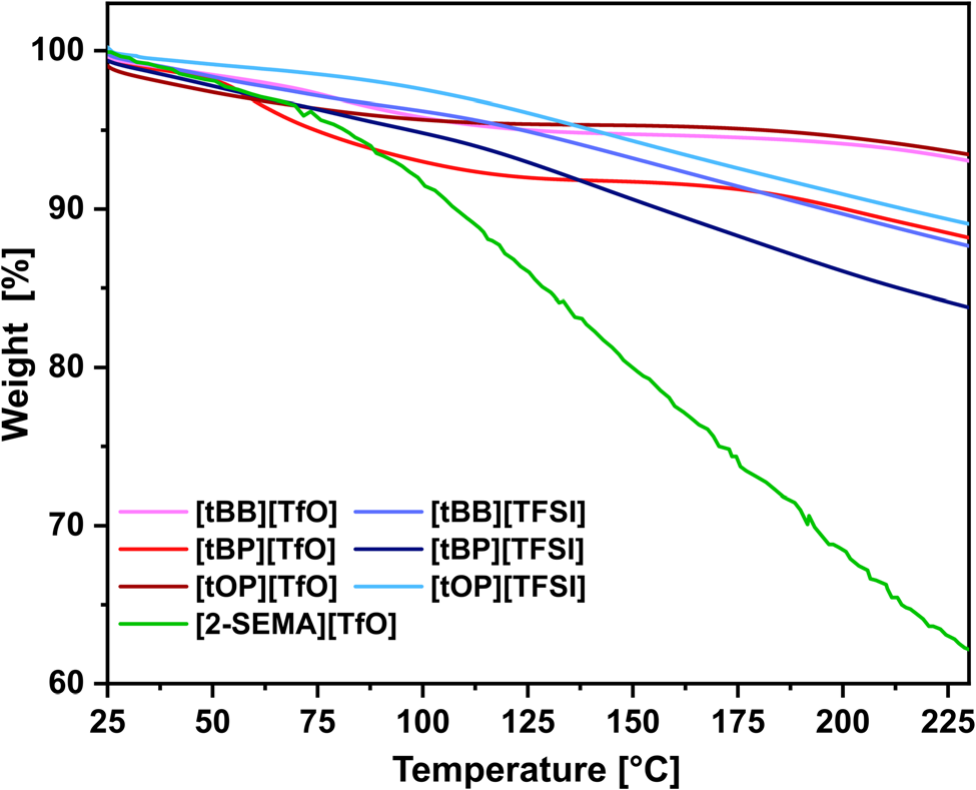
Thermogravimetric analyses (TGA) of the pristine PPILs. [TfO]-based PPILs (red) and [TFSI]-based PPILs (blue) in comparison with [2-SEMA][TfO]^10^ (green) for benchmarking. TGA was carried out from RT to 250 °C with a heating rate of 5 °C min^−1^ under N_2_ atmosphere.

**Fig. 7:**
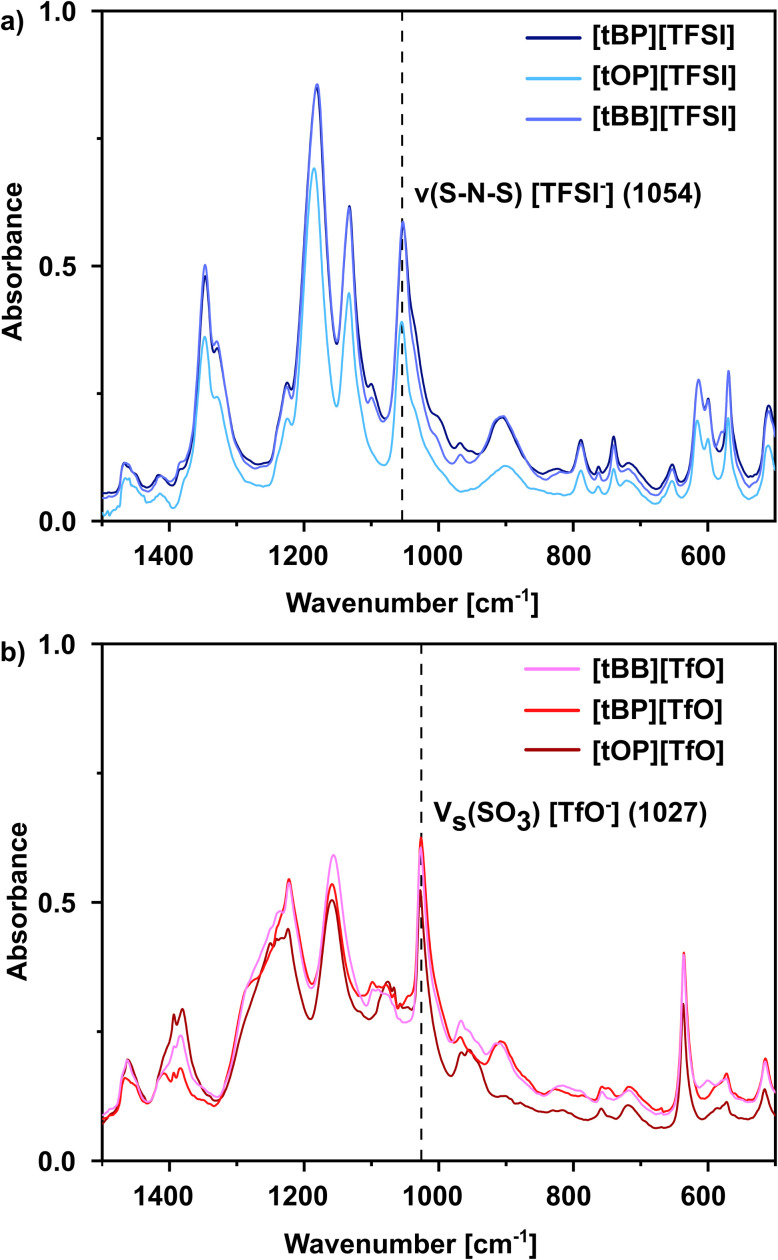
ATR-IR absorbance spectra at 40 °C under air atmosphere; (a) [TFSI]-based PPILs and (b) [TfO]-based PPILs.

Thermal gravimetric analysis (TGA) and IR spectroscopy were employed to study the thermal stability of the novel PPILs, as shown in Fig. 6, 7 and S3. The IR spectra were acquired in addition to the TGA measurements to obtain further evidence on the weight loss of the PPILs. Considering the weight loss profiles of the novel PPILs in Fig. 6, several weight loss steps are present. At temperatures below 100 °C, the mass loss is most likely due to water loss, as the novel PPILs are strongly acidic and hence hygroscopic. The weight-loss steps at temperatures above 100 °C are attributed to the thermal decomposition of the PPILs. Considering the influence of the cation acidity on the thermal stability, the reprotonation of the anion to the conjugated superacid is related to the thermal stability. To understand this concept, the description in the SI in the chapter Formal Considerations: Thermal Stability might be helpful. A [HB][A]-PIL can generally provide proton conductivity *via* a vehicular motion of the cations HB^+^ even at anhydrous states. A cooperative conduction mechanism, as observed, *e.g.* in acidic/basic aqueous systems or neat phosphoric acid, would require at least more than one acidic or basic site on the cation (or anion), an excess of the base B or the presence of an additional amphoteric compound like water to allow proton exchange for long-range charge transfer.^45,46^ For the PPILs shown here, when the reprotonated superacid forms and the temperature exceeds its boiling point, it will leave the PPIL. Affected by cation acidity, molecular weight also influences thermal stability. As the decrease in sample mass is directly proportional to the molecular weight of the cation, which is shown in the form of higher losses of sample mass percentage for the lighter PPILs in Fig. 6, this suggests a thermal stability in the order of [tOP] > [tBB] > [tBP].

Regarding the influence of anions, the major difference in the weight-loss profile is the continuous weight loss observed around the decomposition temperature, *T*_dec_ = 92 °C, for the [TFSI]-based PPILs.^47^ The [TfO]-based PPILs, however, show a plateau in the temperature range between 100 °C and 162 °C. Note that the boiling point of triflic acid (CF_3_SO_3_H, TfOH) is around 160 °C.^48^ From this temperature onward, [TfO]-based PPILs also start to lose mass, following a similar trend to that of the [TFSI]-based PPILs. These thermal decompositions are attributed to an acid–base equilibrium between the sulfonic group on the cation and the anion, resulting in the formation of the conjugated acid. The acid evaporates from the PPIL at its boiling point, leaving behind the zwitterionic deprotonated form of the cation, as shown in eqn (1). This reprotonation effect might give some hints about the acidity of the PPIL cations. Since they are able to reprotonate weak bases such as [TfO] and [TFSI], they might be very strong acids. It might be concluded that the Δp*K*_a_ value between the cation and the strong acid should be comparable to the values, as 15.6 for [2-Sea][TfO] and [2-Sema][TfO].^12,16^

The IR spectra in the “fingerprint” region (400–1500 cm^−1^), shown in Fig. 7a and b, enable the identification of the diagnostic peak attributed to the symmetric stretching *ν*(S–N–S) of the [TFSI] anion, as well as in the case of the [TfO] anion and its symmetric stretching *ν*(SO_3_).^47,48^ These two diagnostic peaks correspond to vibrational modes of the basic functional groups on the anions and are significant for the relative concentrations of deprotonated anions. Therefore, the IR spectra were obtained within the temperature range 30 °C to 130 °C. Fig. S3 shows the peak intensities for [TfO]-based PPILs and [TFSI]-based PPILs, respectively, where an inverse proportionality with temperature is observed. For example, at 40 °C, [tBB][TfO] and [tBB][TFSI] show, respectively, absorbance peak intensities of 0.600 and 0.587. At 100 °C, they show low values of 0.366 and 0.424, respectively. These values indicate a decrease in their concentrations due to conversion to conjugated acids, as assumed in the TGA section. However, the protonation of the [TfO] anion is reversible upon lowering the temperature again. Indeed, comparing the *ν*(SO_3_) intensity peaks at 30 °C in Fig. S4 before and after the ramp-up to 130 °C shows no change in peak height. Since [TfOH] has a higher boiling point than 130 °C, it does not evaporate at the working temperature. The same cannot be confirmed for the [TFSI]-based PPILs. In Fig. S4, by comparing the *ν*(S–N–S) intensity peaks at 30 °C before and after the ramp-up to 130 °C, it is evident that the second collected peak is noticeably lower. Since [TFSIH] has a lower boiling point than 130 °C, a portion of the total amount of [TFSI] anion is lost due to the irreversible evaporation of the conjugated acid.

In light of these findings, the thermal decomposition mechanism profoundly affects the long-term operational stability of IT-PEMFCs. For [TfO]-based PPILs, the thermal stability plateau observed between 100 °C and 162 °C ensures that the electrolyte structure remains intact under standard operating conditions (up to 130 °C). Conversely, for [TFSI]-based PPILs, the continuous weight loss starting at 92 °C and the irreversible loss of the [TFSI] anion, as observed *via* IR, pose a significant risk to durability above 100 °C.

## Conclusions

In this work, six novel phosphonium-based protic ionic liquids (PPILs), based on a combination of 3 different cations and 2 anions, were investigated for their suitability as electrolytes for PEMFC operation above 100 °C: tributyl (3-sulfopropyl) phosphonium [tBP], tributyl (4-sulfobutyl) phosphonium [tBB], and trioctyl (3-sulfopropyl) phosphonium [tOP] with either triflate [TfO] or bistriflimide [TFSI]. It has been reported that differences in cation size strongly influence the properties of PPILs. Firstly, the larger the cation, the lower the dissociation parameter, indicating a negative impact on specific conductivity. Due to the reduced mobility of the larger cations, the vehicular transport mechanism is diminished by inefficient ionic packing. The physicochemical properties of the PPILs revealed oxygen solubility significantly higher than that of analogue sulfoalkylammonium-based PILs investigated in a preceding study. Furthermore, the [TFSI]-based PPILs, on average, exhibit one magnitude higher oxygen diffusion coefficients compared to the [TfO]-based PPILs. This can be attributed to the more apolar, hydrophobic properties of the sulfoalkylphosphonium moiety and of the [TFSI]^−^ anion compared to the [TfO]^−^ anion, respectively. Apolar molecules like O_2_ prefer more apolar environments. Moreover, the anion also influenced the thermal stability, due to the different boiling points of the implemented acids as an anion. It revealed that [TfO]^−^-based PPILs are more suitable as electrolytes for future IT-PEMFCs. Overall, the investigated PPILs showed superior performance compared to ammonium-based PILs (see Table 1).

**Table 1:** Comparison of the decomposition temperature, the oxygen solubility, and the oxygen diffusion coefficients

Electrolytes	*T*_dec_ [°C]	Oxygen solubility [mol cm^−3^]	Oxygen diffusion coefficient [cm^2^ s^−1^]
[TfO]-based PPILs	∼162	∼10^−5^	∼10^−7^
[TFSI]-based PPILs	∼92	∼10^−5^	∼10^−5^
Ammonium-based PILs	∼90	∼10^−6^	∼10^−5^

## Author contributions

T. Bertolin has contributed to conceptualization, methodology, validation, formal analysis, investigation, data curation, writing – original draft, writing – review and editing and visualization. V. Theußl has contributed to conceptualization, validation, investigation, writing – original draft, writing – review and editing, and visualization. R. Leiritz has contributed to validation, formal analysis, investigation, resources, and writing – review and editing. P. Schulz has contributed to conceptualization, methodology, validation, resources, writing – review and editing, supervision, project administration, and funding acquisition. C. Korte has contributed to conceptualization, methodology, software, validation, resources, writing – review and editing, supervision, project administration, and funding acquisition.

## Conflicts of interest

The authors have no conflicts of interest to disclose.

## Supplementary Material

RA-OLF-D6RA04431J-s001

## Data Availability

The data supporting the findings of this study are available from the corresponding author upon reasonable request. Additional supporting information is provided in the supplementary information (SI). Supplementary information is available. See DOI: https://doi.org/10.1039/d6ra04431j.
