# Supplementary material for: Protic phosphonium-based ionic liquids for intermediate temperature polymer electrolyte membrane fuel cells
Source: RSC Adv. 2026 Jul 23. Online ahead of print. doi: 10.1039/d6ra04431j (PMC13394543; doi:10.1039/d6ra04431j)
Supplement: RA-OLF-D6RA04431J-s001 [file RA-OLF-D6RA04431J-s001.pdf]

Supplementary Information (SI) for RSC Advances.
This journal is © The Royal Society of Chemistry 2026

# PROTIC PHOSPHONIUM-BASED PROTIC IONIC LIQUIDS AS ELECTROLYTES FOR IT-PEMFCS

## SUPPLEMENTARY INFO

Tommaso Bertolin,[1,3] Verena Theußl,[1] Rafael Leiritz,[2] Peter Schulz,[2] Carsten Korte[1,3]

[1] Institute of Energy Technologies – Electrochemical Processes Engineering (IET-4), Forschungszentrum Jülich GmbH, 52428 Jülich, Germany

[2] FAU Erlangen University, Department of Chemical and Biological Engineering, 91058 Erlangen, Germany

[3] RWTH Aachen University, Institute of Physical Chemistry, 52074 Aachen, Germany

*Email address of corresponding author: c.korte@fz-juelich.de*

**Table S1:** Reported values for the ionic radii of the PPILs ions.

| Ion | Radius [Å] |
|---|---|
| **[TfO]** | 2.70[1] |
| **[TFSI]** | 3.25[1] |
| **[tBP]** | 4.30[2] |
| **[tBB]** | 4.38[2] |
| **[tOP]** | 5.06[2] |

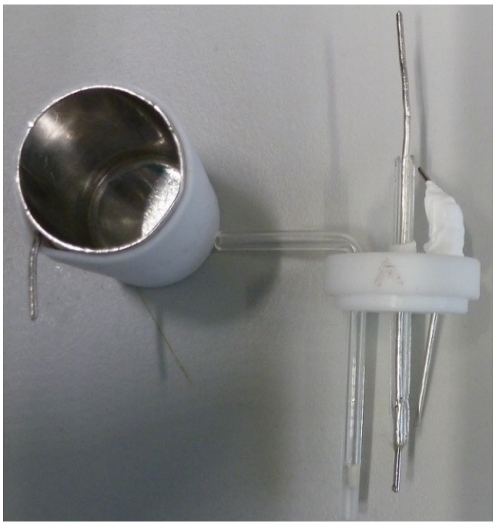

**Figure S1:** 3-electrode set up with Pt wire imbedded in glass, with the counter electrode, a platinum crucible (V= 5 mL) embedded in a Teflon insulator.

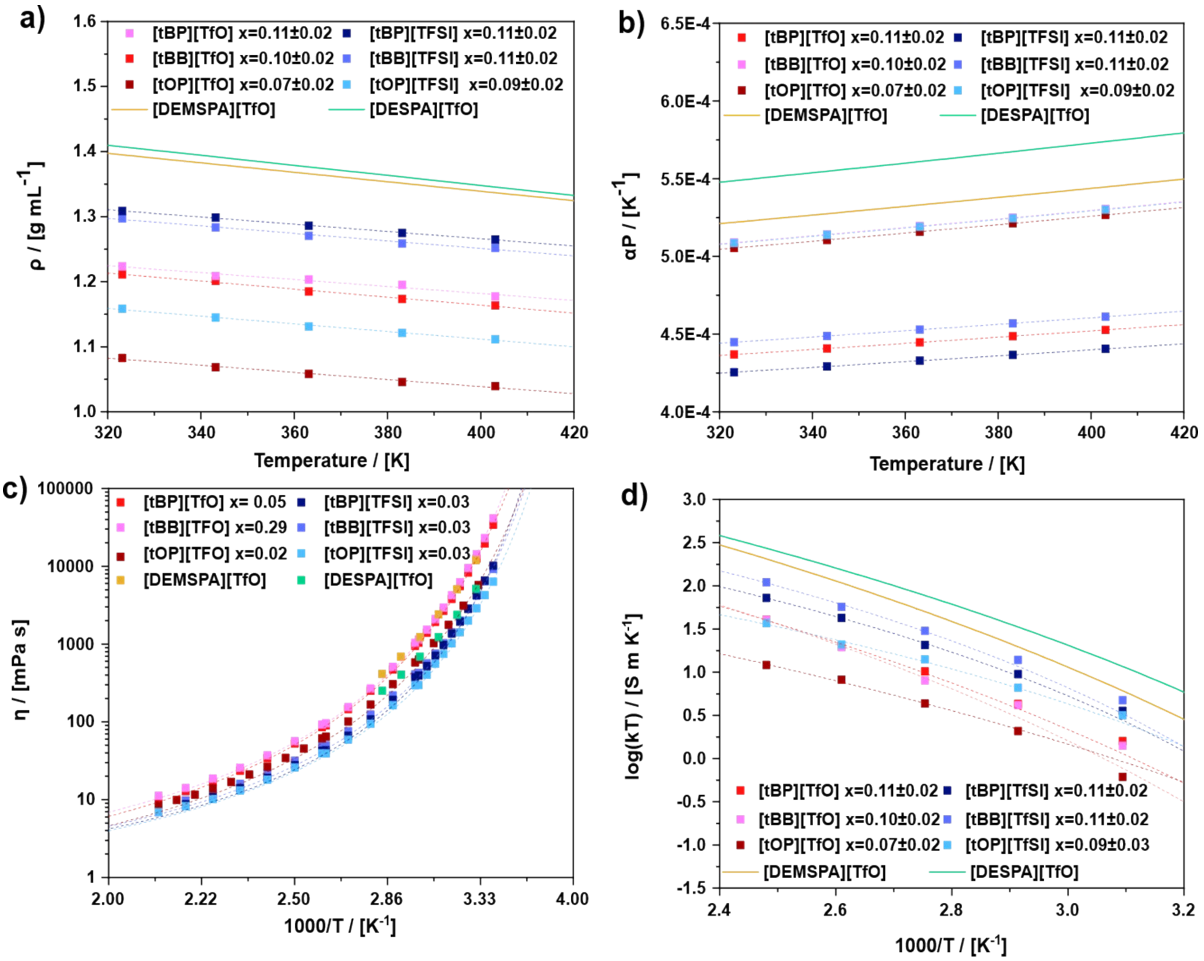


**Figure S2:** Physicochemical properties of the novel PPILs as a function of temperature: **a)** Density, **b)** Isobaric thermal expansivity, **c)** Dynamic viscosity and **d)** specific conductivities. The density data were obtained under ambient conditions at low water content (x=0.1 water molar ratio) with a pycnometer by increasing the temperature and checking the water content after each density measurement.

**FORMAL CONSIDERATIONS: IONIC (PROTIC) CONDUCTION IN VISCOUS LIQUID ELECTROLYTES**

A [HB][A]-PIL can generally provide proton conductivity *via* a vehicular motion of the cations $HB^+$ even at anhydrous states. A cooperative conduction mechanism, as observed *e.g.* in acidic/basic aqueous systems or neat phosphoric acid, would require at least more than one acidic or basic sites on the cation (or anion), an excess of the base B or the presence of an additional amphoteric compound like water to allow proton exchange for long-range charge transfer.[3,4]

In a viscous liquid electrolyte system close to the glass temperature Tg, ionic motion and local viscous flows are strongly correlated. A simple model, assuming ions as hard spheres moving in a viscous medium and an equilibrium between the forces due to viscous friction and the dragging electric field, yields the Stokes-Einstein in Eq. (4).

$$D_i = \frac{k_B T}{6\pi\eta r_i} \quad \text{and} \quad \sigma_i = \frac{(z_i e)^2}{6\pi\eta r_i} c_i \qquad \text{(S1)}$$

This simple model connects the (self-) diffusion coefficient $D_\mathrm{i}$ of a species $i$, respectively its partial conductivity $\sigma_i$ *via* the Nernst-Einstein equation, with its ionic radius $r_i$, concentration $c_i$, and the dynamic viscosity $\eta$ of the electrolyte solution. [5] The Boltzmann constant is denoted with $k_\mathrm{B}$, the temperature with $T$ and the elementary charge with $e$.

In a more elaborated model the motion of an ion in a viscous electrolyte close to the glass temperature can be related to the probability that a sufficiently large free volume is formed close to an ion caused by thermal motion. This probability is rate-limiting. The total free volume is increasing with temperature due to thermal expansion. Thus, when reaching a characteristic temperature $T_0$, which should be related to the glass temperature, the free volume vanishes, and the ionic conductivity should tend to zero. Generally, the coupling between ionic motion and local viscous flow will result in a non-Arrhenius behaviour of ionic conductivity and viscosity. This behaviour can be empirically described by the VFT equation for the viscosity $\eta$ in Eq. (5) and (total) ionic conductivity σ in Eq. (6) of the electrolyte solution:

$$\sigma = \sigma_0 exp\left[-\frac{B_\sigma}{R\,(T - T_0)}\right] \qquad \text{(S2)}$$

$$\eta = \eta_0 \exp\left[\frac{B_\eta}{R\,(T - T_0)}\right] \qquad \text{(S3)}$$

Hereby, $B_\sigma$ and $B_\eta$ are the pseudo-activation energies of the ionic transport and the viscous flow, $\sigma_0$ and $\eta_0$ are the pre-exponential factors, and R is the universal gas constant. When $T$ is large compared to $T_0$, Eq. (5) and (6) turn to the Arrhenius law, respectively to the Arrhenius-Andrade law:

$$\sigma = \frac{\sigma_0}{T} exp\left[ - \frac{E_{a,\sigma}}{RT} \right] \tag{S4}$$

$$\eta = \eta_0 \exp\left[ \frac{E_{a,\eta}}{RT} \right] \tag{S5}$$

The Arrhenius-type activation energies are denoted with $E_{a,\sigma}$ and $E_{a,\eta}$. At elevated temperatures, far from $T_0$, the rate-limiting step for ionic motion is the jump to a neighbouring position.

Assuming a strong correlation between ionic motion and local viscous flow according to the Stokes-Einstein equation, see Eq. (4), an expression for the total conductivity σ of a binary electrolyte can be derived. To obtain an expression independent of the concentration, the molar conductivity $\Lambda$ is introduced in Eq. (9):

$$\Lambda = \frac{\sigma}{c} \quad \text{and} \quad \sigma = \sigma_+ + \sigma_- \tag{S6}$$

Hereby, $\sigma_+$ and $\sigma_-$ are the partial conductivities of the cation and anion. If there is an incomplete dissociation, the concentrations of $c_+$ and $c_-$ depend on the dissociation degree $\alpha_D$ as shown in Eq. (S7):

$$c_+ = \alpha_D \nu_+ c \quad \text{and} \quad c_- = \alpha_D \nu_- c \tag{S7}$$

The stoichiometric factors are denoted with $\nu_+$ and $\nu_-$. Complete dissociation is indicated by $\alpha_D$ equal to 1. This yields an expression for the product of the dynamic viscosity η and the molar conductivity $\Lambda$, only dependent on the ionic radii, the stoichiometric factors and the dissociation degree as parameters and can be merged in a matter-specific constant $C$ (Walden rule), in Eq. (S8):

$$\Lambda\eta = \frac{e^2 N_A}{6\pi} \left( \frac{z_+^2 \nu_+}{r_+} + \frac{z_-^2 \nu_-}{r_-} \right) \alpha_D = C \tag{S8}$$

Thus, plotting the logarithm of $\Lambda$ *vs.* the logarithm of 1/η (Walden plot) should yield a linear relation with a slope of 1, independent of concentration and temperature:

$$\lg \Lambda = \lg C + \lg \frac{1}{\eta} \quad \text{and} \quad C = \frac{e^2 N_A}{6\pi} \left( \frac{z_+^2 \nu_+}{r_+} + \frac{z_-^2 \nu_-}{r_-} \right) \alpha_D \tag{12}$$

However, the constant $C$ is a function of the dissociation degree, *i.e.* if α is smaller than 1, the values are shifted down the ordinate but retain a slope of 1. Conductivity data is usually compared in the literature to a dilute KCl solution, where complete dissociation can be assumed, and only vehicular transport is possible ("KCl line"). Considering the ionic radii of $K^+$ and $Cl^-$ of 1.25 Å and 1.21 Å, a value of 1,33 · $10^{-5}$ S $m^2$ $mol^{-1}$ Pa s can be calculated for $C_{KCl}$. Using the unit S $cm^2$ $mol^{-1}$ for the molar

conductivity and Poise (10 P = 1 Pa s) for the viscosity, $\lg C_{\mathrm{KCl}}$ has a value of only 0.12. Thus, the "KCl line" hits approximately the origin of such a plot with a slope of 1. [6]

To compare different electrolytes with various degrees of dissociation and thus different ionicities, a used approach is to normalise the dynamic viscosity by the ionic radii. The intercept with the ordinate should only be dependent on simple physical constants and the degree of dissociation $\alpha_D$, as shown in Eq. (S):

$$\lg \Lambda = \lg C' + \lg \left[ \frac{1}{\eta} \left( \frac{z_+^2 v_+}{r_+} + \frac{z_-^2 v_-}{r_-} \right) \right] \quad \text{and} \quad C' = \frac{e^2 N_A}{6\pi} \alpha_D \tag{S9}$$

In the case of a 1:1 electrolyte, a plot of $\lg \Lambda$ *vs.* $\lg [1/\eta \, (1/r_+ + 1/r_-)]$ has to be performed. The closer the ordinate intercept is to $\lg (e^2 N_A/6\pi)$, the more the ionic species are fully dissociated.[5,7,8] The values of the anionic radii are those determined in the literature, while the values of the cationic radii were determined by a Van-der-Waals volume calculation proposed in the literature.

All considerations above are based on a strong coupling between ionic motion and local viscous flow. Regarding Eq. (5) and (6), this implies that the pseudo-activation energies $B_\sigma$ and $B_\eta$ are identical, as the same process is rate-limiting. A cooperative transport mechanism for protons overrides the strong coupling of $B_\sigma$ and $B_\eta$, as no local viscous fluxes of the particles in the neighbourhood are necessary, but only local rearrangements of the ions by rotation to transfer the proton. This effect results in a decreased pseudo-activation energy $B_\sigma$ for ionic transport compared to $B_\eta$ for viscous flow, as described in Eq. (S10):

$$B_\sigma = \alpha B_\eta \tag{S10}$$

The factor α is a measure of the decoupling of ionic transport and local viscous flux and should be smaller than 1 ($\alpha \leq 1$). In this case, the original Walden rule in Eq. (S8) is modified to the fractional Walden rule law, as shown in Eq. (S11) and Eq. (S12):

$$\Lambda \eta^\alpha = C'' \tag{S11}$$

$$\lg \Lambda = \lg C'' + \alpha \lg \frac{1}{\eta} \tag{S12}$$

In the case of proton conduction, a value of $\alpha = 1$ indicates that the ionic transport is solely caused by a vehicular mechanism. With decreasing α, *i.e.* $\alpha < 0$, an increasing share of cooperative transport is present.

**Table S2:** Extrapolated VFT fitting parameters from the Dynamic Viscosity and the Specific Conductivity

| PPIL | Dynamic viscosity | | Total Conductivity | | $\alpha_D$ |
|---|---|---|---|---|---|
| | $B_\eta \left[\frac{kJ}{mol}\right]$ | $T_0$ **[K]** | $B_\sigma \left[\frac{kJ}{mol}\right]$ | $T_0$ **[K]** | **T=90 °C** |
| **[tBP][TfO]** | 12.0 ±0.9 | 183.3 ± 7.2 | 11.4 ±0.5 | 177.3 ±5.2 | 1.05 ±0.04 |
| **[tBB][TfO]** | 11.3 ±1.0 | 189.2 ± 7.6 | 11.3 ±0.5 | 187.5 ±5.4 | 0.98 ±0.03 |
| **[tOP][TfO]** | 11.9 ±1.9 | 177.8 ± 7.8 | 9.0 ±0.5 | 167.3 ±7.2 | 0.35 ±0.03 |
| **[tBP][TFSI]** | 8.6 ±1.8 | 201.2 ± 7.2 | 6.4 ±0.4 | 214.6 ±1.9 | 0.79 ±0.01 |
| **[tBB][TFSI]** | 9.5 ±1.9 | 192.8 ± 7.4 | 6.9 ±0.1 | 214.8 ±4.9 | 1.21 ±0.02 |
| **[tOP][TFSI]** | 9.4 ±0.9 | 190.9 ± 8.5 | 5.8 ±0.5 | 205.4 ±7.2 | 0.72 ±0.01 |

**Table S3:** Reported values of the anion/cation radii ratio and the corresponding fitting value for the Walden plot

| **PPIL** | $(\frac{r^-}{r^+})$ | $\log C'$ | $\alpha$ | $\frac{B_\sigma}{B_\eta}$ |
|---|---|---|---|---|
| **[tBP][TfO]** | 0.63 | -0.28 | 0.94 | 0.95 |
| **[tBB][TfO]** | 0.62 | -0.19 | 1.01 | 1.00 |
| **[tOP][TfO]** | 0.53 | -0.88 | 0.76 | 0.76 |
| **[tBP][TFSI]** | 0.76 | -0.42 | 0.74 | 0.75 |
| **[tBB][TFSI]** | 0.74 | -0.26 | 0.73 | 0.72 |
| **[tOP][TFSI]** | 0.64 | -0.52 | 0.62 | 0.61 |

## FORMAL CONSIDERATIONS: THERMAL STABILTY

The activities $a_{HB^+}$, $a_{A^-}$, $a_{HA}$ and $a_B$ of the products and educts in Eq. (S13), respectively the concentrations, depend on the constant $K$ of the corresponding mass action law:

$$K = \frac{a_{HB^+}\, a_{A^-}}{a_{HA}\, a_B} \tag{S13}$$

Neglecting the strict validity of the Brønsted acidity constants only for diluted aqueous systems, the equilibrium constant $K$ in Eq. (2) can be expressed as the ratio $K_{a,HA}/K_{a,HB^+}$ in Eq. (S14) of the acidity constants of the conjugated acid HA of the anion and the cation $HB^+$:

$$K = K_{a,HA} \cdot \frac{1}{K_{a,HB^+}} = \frac{a_{A^-}\, a_{H_3O^+}}{a_{HA}\, a_{H_2O}} \cdot \frac{a_{HB^+}\, a_{H_2O}}{a_B\, a_{H_3O^+}} \tag{S14}$$

Thus, a very high $\Delta pK_a$ value ensures negligible small concentrations of the free base B and acid HA in the equilibrium. As the free neutral components are potentially volatile, this will determine the thermal stability of a [HB][A]-PIL.[6] The ΔpKa of the low acidic PIL [Dema][TfO] with a value of 25.3 is very high. It shows the onset of thermal decomposition at around 250 °C, which precedes any boiling process. On the other hand, the $\Delta pK_a$ of the high acidic PIL [2-Sema][TfO] has only a value of 15.6, resulting in a decomposition already at 160°C, which corresponds to the boiling point of triflic acid [TfOH].[9]

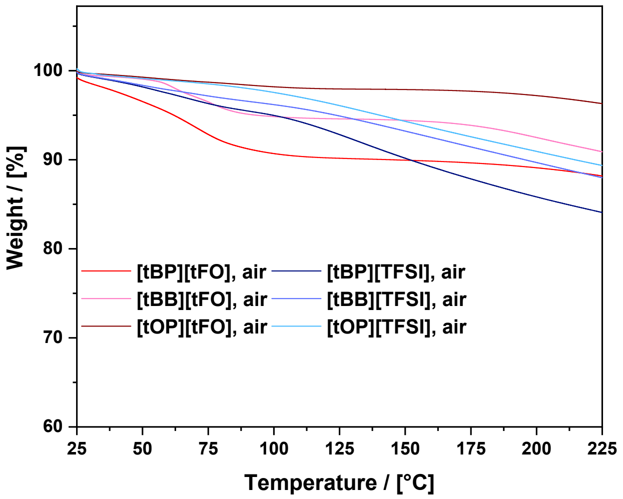


**Figure S3**: Thermogravimetric analyses (TGA) of the pristine PPILs. [TfO]-based PPILs (blue) and [TFSI]-based PPILs (red) in comparison with [2-SEMA][TfO][10] (green) for benchmarking. TGA was carried out from RT to 250 °C with a heating rate of 5 °C min-1 under air atmosphere

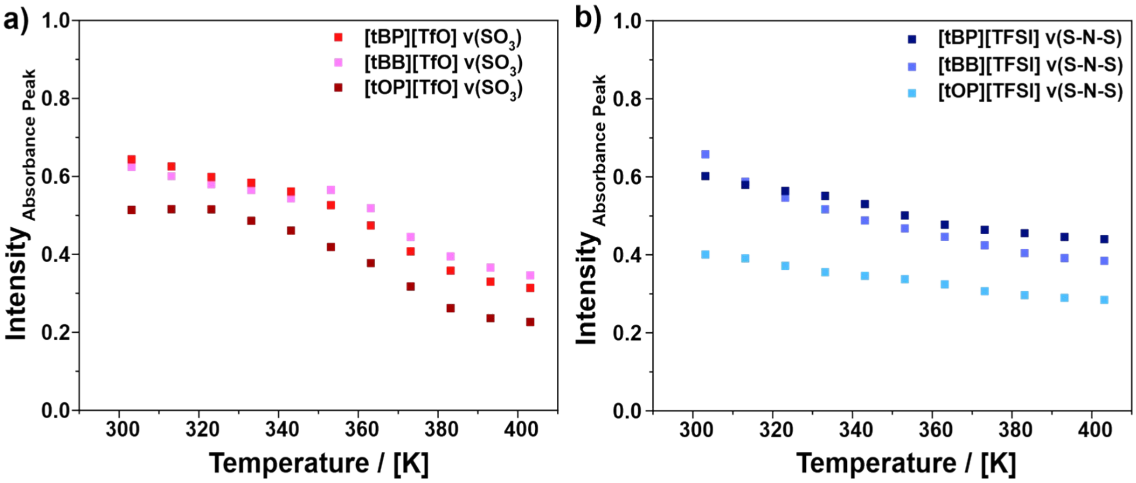


**Figure S4:** Intensities of the unique ATR-IR peaks of **(a)** [TfO]-based PPILs (v($SO_3$) @ 1027 $cm^{-1}$) and of **(b)** [TFSI]-based PPILs (v(S-N-S) @ 1050 $cm^{-1}$) in dependence of increasing temperature. The ATR-IR spectra are collected within a temperature range of 30-130 °C, with a scan rate v=5 °C/min under ambient conditions.

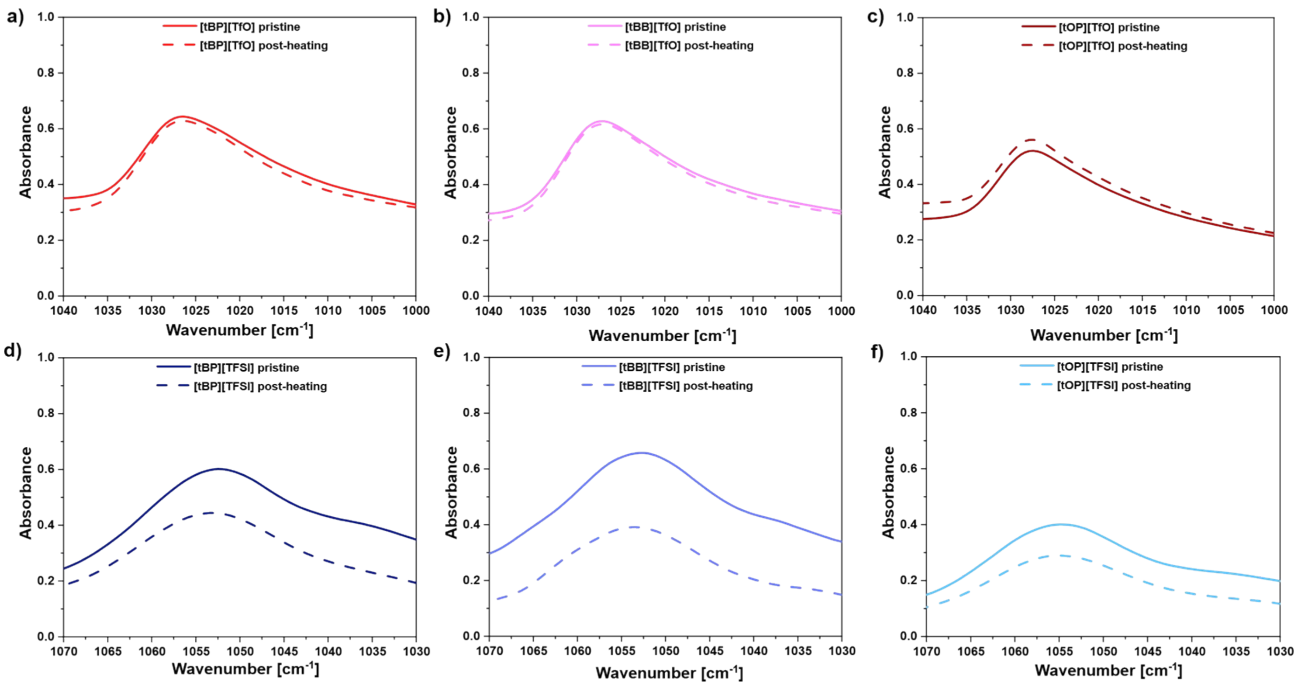


**Figure S5**: ATR-IR absorbance peaks for the unique peaks of (**a-c**) [TfO]-based PPILs (v($SO_3$) @ 1027 $cm^{-1}$) and of (**d-e**)[TFSI]-based PPILs (v(S-N-S) @ 1050 $cm^{-1}$), recorded at 30 °C in the pristine state (solid line) and after the temperature program until 130°C (dashed line). The ATR-IR spectra are collected within a temperature range of 30-130 °C, with a scan rate v=5 °C/min under ambient conditions.

**SUPPORTING INFORMATION BYBLIOGRAPHY**


1. Ue, M. Mobility and Ionic Association of Lithium and Quaternary Ammonium Salts in Propylene Carbonate and γ-Butyrolactone. *J. Electrochem. Soc.* **141**, 3336–3342 (1994).
2. Zhao, Y. H., Abraham, M. H. & Zissimos, A. M. Fast calculation of van der Waals volume as a sum of atomic and bond contributions and its application to drug compounds. *Journal of Organic Chemistry* **68**, 7368–7373 (2003).
3. Umebayashi, Y., Han, J. & Watanabe, H. Toward New Ion Conductive Liquids via Ionic Liquids. *The Chemical Record* **23**, e202200302 (2023).
4. Feng, G. *et al.* Free and Bound States of Ions in Ionic Liquids, Conductivity, and Underscreening Paradox. *Phys. Rev. X* **9**, 21024 (2019).
5. Sutherland, W. LXXV. A dynamical theory of diffusion for non-electrolytes and the molecular mass of albumin. *The London, Edinburgh, and Dublin Philosophical Magazine and Journal of Science* **9**, 781–785 (1905).
6. Yoshizawa, M., Xu, W. & Angell, C. A. Ionic Liquids by Proton Transfer: Vapor Pressure, Conductivity, and the Relevance of ΔpKa from Aqueous Solutions. *J. Am. Chem. Soc.* **125**, 15411–15419 (2003).
7. MacFarlane, D. R. *et al.* On the concept of ionicity in ionic liquids. *Physical Chemistry Chemical Physics* **11**, 4962–4967 (2009).
8. Ue, M. Mobility and Ionic Association of Lithium and Quaternary Ammonium Salts in Propylene Carbonate and γ-Butyrolactone. *J. Electrochem. Soc.* **141**, 3336 (1994).
9. Suo, Y. *et al.* Binary Mixtures of Proton-Conducting Ionic Liquids as Electrolytes for Medium-Temperature Polymer Electrolyte Membrane Fuel Cells. *The Journal of Physical Chemistry C* **125**, 21588–21594 (2021).
